# Reproducibility of Diagnostic Criteria for Ventricular Neurocysticercosis

**DOI:** 10.4269/ajtmh.17-0724b

**Published:** 2017-12-06

**Authors:** Javier A. Bustos, Héctor H. García, Oscar H. Del Brutto

**Affiliations:** Center for Global Health – Tumbes, Universidad Peruana Cayetano Heredia, Cysticercosis Unit, Instituto Nacional de Ciencias Neurológicas, Lima, Peru Department of Microbiology, Universidad Peruana Cayetano Heredia, Cysticercosis Unit, Instituto Nacional de Ciencias Neurológicas, Lima, Peru E-mails: javier.bustos@jhu.edu and hgarcia1@jhu.edu; School of Medicine, Universidad Espíritu Santo – Ecuador, Guayaquil, Ecuador E-mail: oscardelbrutto@hotmail.com

Dear Sir,

In the letter “Reproducibility of Diagnostic Criteria for Ventricular Neurocysticercosis,”^1^ the authors manifest their unhappiness with the results of the systematic review we conducted to assess reliability of the revised Del Brutto’s set of diagnostic criteria for ventricular neurocysticercosis (NCC), mainly concerning specificity.^2^

Specificity is the ability of a test to appropriately detect as negative a condition that does not exist in a given patient. According to the revised Del Brutto’s set of diagnostic criteria for NCC,^3^ only four of the 41 reviewed patients with other ventricular infections would be erroneously diagnosed as definitive NCC.^2^ These four false-positive diagnoses resulted from the presence of two major neuroimaging findings (ventricular cystic lesion plus additional parenchymal lesions) together with clinical/exposure criteria.^3^ None of the 41 cases had any absolute criteria for NCC, nor any confirmative neuroimaging criteria, and as such no other definitive diagnoses of NCC would be made using our criteria. This means that for this set we found a specificity of 90.2% (95% confidence interval [CI]: 75.9–96.8%), as detailed in our systematic review.^2^ We don’t understand how specificity was calculated as 31.7% or 55% by Fleury et al. Perhaps they included “probable” cases as positively diagnosed.

According to the modified set of criteria for NCC used to determine the specificity for our set, a definitive diagnosis of extraparenchymal NCC is established in patients presenting with subarachnoid or intraventricular cysts without scolex, associated with at least two of the following: 1) hydrocephalus, 2) inflammatory cerebrospinal fluid (CSF), 3) positive CSF immunological tests (enzyme-linked immunosorbent assay (ELISA) or enzyme-linked immunotransfer blot (EITB), and 4) presence of single or multiple calcifications or parenchymal vesicular or degenerating cysts.^4^ The poor specificity of this categorization is obvious because any patient with an intraventricular cyst associated with hydrocephalus and an inflammatory CSF would be characterized as definitive NCC. Both hydrocephalus and inflammatory abnormalities in the CSF are unspecific, and many patients with other infections requiring different management might be misdiagnosed as NCC.^5^ Indeed, 25 of the 41 cases with proven non-cysticercotic ventricular lesions that we analyzed in our systematic review had either ventricular cystic or granulomatous lesions (with a cystic component) without scolex associated with hydrocephalus and inflammatory CSF or with concomitant parenchymal lesions.^4^ These 25 false-positive cases thus lower the specificity to 39% (95% CI: 24.6–55.5%). It is difficult to understand how specificity, as calculated by Fleury et al, was 78.1% or 82.5% in spite of the categorization of these 25 cases. Again, one possibility is that their case review was not blind, and perhaps they did not categorize lesions with cystic contents as cysts. Relevant information on the 25 false-positive cases is summarized in Table 1.

**Table 1:** Cases with non-cysticercotic–related ventricular cystic lesions or granulomas, where the diagnosis is definitive cysticercosis based on the modified set of diagnostic criteria proposed by Carpio et al.^4^

Reference	Ventricular cystic lesion or granuloma*	Other imaging findings	Hydrocephalus	CSF analysis	Definitive diagnosis
Acta Neurochir 2004;146:1151	Yes	–	Yes	Inflammatory	Tuberculoma
AJNR 1996;17:110	Yes	–	Yes	Inflammatory	Cryptococcosis
AJNR 2002;23:273	Yes	Parenchymal cyst no scolex	Yes	Inflammatory	Cryptococcosis
BMJ Case Rep 2014;bcr2014-203837	Yes	–	Yes	Inflammatory	Tuberculoma
Br J Radiol 2010;83-e14	Yes	–	Yes	Inflammatory	Cryptococcosis
Case Rep Clin Med 2013;2:81	Yes	–	Yes	Inflammatory	Tuberculoma
Case Rep Neurol 2015;7:156	Yes	–	Yes	Inflammatory	Pyogenic abscess
Clin Infect Dis 1993;16:435	Yes	–	Yes	Inflammatory	Pyogenic abscess
Indian J Pathol Microbiol 2008;51:553	Yes	Parenchymal cyst no scolex	–	Inflammatory	Cryptococcosis
Indian J Tuberc 2014;61:166	Yes	–	Yes	Inflammatory	Tuberculoma
Indian Pediatr 2011;48:161	Yes	–	Yes	Inflammatory	Tuberculoma
J Comput Assist Tomogr 1993;17:547 (case 1)	Yes	–	Yes	Inflammatory	Cryptococcosis
J Comput Assist Tomogr 1993;17:547 (case 2)	Yes	–	Yes	Inflammatory	Cryptococcosis
J Craniofacial Surg 2010;21:1291	Yes	–	Yes	Inflammatory	Aspergillosis
J Neuroradiol 2008;35:63	Yes	–	Yes	Inflammatory	Tuberculoma
Mayo Clin Proc 1999;74:803	Yes	–	Yes	Inflammatory	Histoplasmosis
Med Mycol Case Rep 2015;10:18	Yes	–	Yes	Inflammatory	Rhizopus abscess
Neurol India 1999;47:327	Yes	–	Yes	Inflammatory	Tuberculoma
Neurol India 2014;62:73	Yes	Parenchymal cyst no scolex	Yes	–	Tuberculoma
Neurol Med Chir (Tokyo) 2002;42:501	Yes	–	Yes	Inflammatory	Tuberculoma
Neuropathology 2014;34:210	Yes	–	Yes	Inflammatory	Cryptococcosis
Neuroradiology 1993;35:149	Yes	Parenchymal cyst no scolex	Yes	–	Hydatid disease
Neuroradiology 2003;45:908	Yes	–	Yes	Inflammatory	Pyogenic abscess
Pediatr Neurosurg 2017;52:93	Yes	–	Yes	Inflammatory	Tuberculoma
Surg Neurol 2007;67:647	Yes	–	Yes	Inflammatory	Cryptococcosis

* Granuloma with cystic component.

## References

[1] Fleury A Carpio A Romo ML San-Juan D Sander JW, 2017 Reproducibility of diagnostic criteria for ventricular neurocysticercosis. Am J Trop Med Hyg 97: 1952. 29187272 10.4269/ajtmh.17-0724a PMC5805080

[2] Bustos JA Garcia HH Del Brutto OH, 2017 Reliability of diagnostic criteria for neurocysticercosis for patients with ventricular cystic lesions or granulomas: a systematic review. Am J Trop Med Hyg 97: 653–657. 28820689 10.4269/ajtmh.17-0069 PMC5590602

[3] Del Brutto OH Nash TE White AC Jr Rajsherkhar V Wilkins PP Sing G Vasquez CM Salgado P Gilman RH Garcia HH, 2017 Revised diagnostic criteria for neurocysticercosis. J Neurol Sci 372: 202–210. 28017213 10.1016/j.jns.2016.11.045

[4] Carpio A 2016 New diagnostic criteria for neurocysticercosis: reliability and validity. Ann Neurol 80: 434–442. 27438337 10.1002/ana.24732 PMC5053253

[5] Gilman RH, 2016 Diagnostic criteria for neurocysticercosis – a difficult update. Nat Rev Neurol 12: 560–561. 27677966 10.1038/nrneurol.2016.145

